# Transcriptome and DNA Methylation Profiles of Mouse Fetus and Placenta Generated by Round Spermatid Injection

**DOI:** 10.3389/fcell.2021.632183

**Published:** 2021-03-16

**Authors:** Haibo Zhu, Hao Sun, Dawei Yu, Tianda Li, Tang Hai, Chao Liu, Ying Zhang, Yurong Chen, Xiangpeng Dai, Ziyi Li, Wei Li, Ruizhi Liu, Guihai Feng, Qi Zhou

**Affiliations:** ^1^Center of Reproductive Medicine, Center of Prenatal Diagnosis, First Hospital, Jilin University, Changchun, China; ^2^State Key Laboratory of Stem Cell and Reproductive Biology, Institute of Zoology, Chinese Academy of Sciences, Beijing, China; ^3^Key Laboratory of Organ Regeneration and Transplantation of the Ministry of Education, First Hospital, Jilin University, Changchun, China; ^4^Institute of Stem Cell and Regenerative Medicine, Chinese Academy of Sciences, Beijing, China; ^5^University of Chinese Academy of Sciences, Beijing, China

**Keywords:** round spermatid injection, transcriptome, DNA methylation, imprinted gene, fetus, placenta

## Abstract

Low birth efficiency and developmental abnormalities in embryos derived using round spermatid injection (ROSI) limit the clinical application of this method. Further, the underlying molecular mechanisms remain elusive and warrant further in-depth study. In this study, the embryonic day (E) 11.5 mouse fetuses and corresponding placentas derived upon using ROSI, intracytoplasmic sperm injection (ICSI), and natural *in vivo* fertilized (control) embryos were collected. Transcriptome and DNA methylation profiles were analyzed and compared using RNA-sequencing (RNA-seq) and whole-genome bisulfite sequencing, respectively. RNA-seq results revealed similar gene expression profiles in the ROSI, ICSI, and control fetuses and placentas. Compared with the other two groups, seven differentially expressed genes (DEGs) were identified in ROSI fetuses, and ten DEGs were identified in the corresponding placentas. However, no differences in CpG methylation were observed in fetuses and placentas from the three groups. Imprinting control region methylation and imprinted gene expression were the same between the three fetus and placenta groups. Although 49 repetitive DNA sequences (RS) were abnormally activated in ROSI fetuses, RS DNA methylation did not differ between the three groups. Interestingly, abnormal hypermethylation in promoter regions and low expression of *Fggy* and *Rec8* were correlated with a crown-rump length less than 6 mm in one ROSI fetus. Our study demonstrates that the transcriptome and DNA methylation in ROSI-derived E11.5 mouse fetuses and placentas were comparable with those in the other two groups. However, some abnormally expressed genes in the ROSI fetus and placenta warrant further investigation to elucidate their effect on the development of ROSI-derived embryos.

## Introduction

Since the first successful use of *in vitro* fertilization (IVF) in Britain in 1978 (Steptoe and Edwards, 1978), rapid advances in assisted reproductive technology (ART) have benefited millions of families worldwide (Niederberger et al., 2018; De Geyter, 2019). However, concerns about the health of babies conceived using ART remain (Lazaraviciute et al., 2014; Kawwass and Badell, 2018; Hattori et al., 2019), especially since 1992, when intracytoplasmic sperm injection (ICSI) was first employed to produce offspring (Palermo et al., 1992). Compared with IVF, there are no obvious congenital malformations or epigenetic disorders in children conceived through ICSI (Palermo et al., 2015), and the technique is widely used worldwide. However, it is too early to conclude that this technology is safe because the oldest individual produced using ICSI is less than 30 years of age. Notably, after the successful injection of mature sperm into the oocyte to produce offspring in animals and humans (Kimura and Yanagimachi, 1995a), the microinjection of immature germ cells, such as round spermatids, into oocytes has been evaluated (Ogura et al., 2005). Round spermatids are precursors of mature spermatozoa, and the transformation of round spermatids into mature spermatozoa involves the replacement of histones in chromatin with arginine-rich protamine, acrosome formation, and flagellum formation, resulting in the attainment of motility (Yanagimachi, 2005).

Although mice and other species were produced using round spermatid injection (ROSI) as early as 1995 (Kimura and Yanagimachi, 1995b; Tesarik and Mendoza, 1996; Hirabayashi et al., 2009), the clinical application of this approach has been limited owing to its inefficiency and safety considerations (ASRM and SART, 2008; Gross et al., 2020), including low blastocyst percentage and high abortion rates (Ghazzawi et al., 1999; Sousa et al., 2002; Ogonuki et al., 2003). Many unresolved issues concerning this technology remain. First, how the artificial method used to activate eggs injected with round spermatids affects embryonic development is not fully understood (Kishigami et al., 2004). Second, it is unclear whether round spermatids and mature sperm share the same imprinting state (Shamanski et al., 1999; Sasaki and Matsui, 2008; Chen et al., 2018). Third, in animal studies, the early division abnormality and aneuploidy rates of ROSI embryos were higher than those of embryos fertilized via injection of mature sperm (Ogonuki et al., 2005; Yamagata et al., 2009). Lastly, early epigenetic abnormalities are frequently observed in the male genome from round spermatids (Kishigami et al., 2006; Kong et al., 2017), and some ROSI-derived embryos fail to undergo active DNA demethylation, leading to abnormally sized fetuses at embryonic day (E) 11.5 (Kurotaki et al., 2015). Therefore, the Practice Committee of the American Society for Reproductive Medicine has classified ROSI as an experimental technology (ASRM and SART, 2008). Importantly, Tanaka et al. (2015) optimized the ROSI procedure and successfully produced 14 healthy children (Tanaka et al., 2015). Three years later, the same team reported that children conceived using ROSI are not at an increased risk of developing congenital malformations (Tanaka et al., 2018), an observation that seems promising for the clinical application of ROSI.

Mid-pregnancy–following ROSI fertilization–is a crucial stage for investigations on the molecular mechanism underlying abnormal development. No comprehensive studies have been conducted on fetuses and placentas at mid-pregnancy–following ROSI–due to material limitations and methodological constraints. However, some critical questions about ROSI remain. For example, are the gene expression patterns of the fetus and placenta derived upon fertilization with round spermatids similar to those of embryos derived upon fertilization with mature sperm? Are epigenetic modifications different between embryos derived upon fertilization with ROSI and ICSI? The development of whole-genome bisulfite sequencing (WGBS) technology has enabled genome-wide DNA methylation analyses (Zhang et al., 2017). In this study, we used the mouse as a model to study the transcriptome and DNA methylation profiles in E11.5 fetuses and placentas derived upon fertilization with ROSI. Our results provide insights into the mechanisms responsible for the high frequency of abnormalities arising as a result of ROSI.

## Materials and Methods

The study was mainly completed at the First Hospital of Jilin University. However, E11.5 mouse fetus sample collection was conducted at the State Key Laboratory of Stem Cell and Reproductive Biology, Institute of Zoology, Chinese Academy of Sciences. Experiment-related operations followed the same protocol, with some exceptions.

### Animals

B6D2F1 (C57BL/6 × DBA/2), C57BL/6, and ICR background mice were purchased from Beijing Vital River Laboratory Animal Technologies Co., Ltd., (Beijing, China). All mice were housed in the Laboratory Animal Center of the First Hospital of Jilin University. The light period was 8 am to 8 pm, and mice were provided *ad libitum* access to food and water.

### Preparation of Oocytes

Pregnant mare serum gonadotropin (PMSG) (7.5 IU; Ningbo Second Hormone Company, Ningbo, China) was injected into 6- to 10-week-old female B6D2F1 mice. After 48 h, 7.5 IU human chorionic gonadotropin (HCG) (Ningbo Second Hormone Company) was injected into the same mice. Then, 14 h after the HCG injection, mice were euthanized, and oocyte corona cumulus complexes (OCCOs) were collected from the ampulla of the fallopian tube. OCCOs were placed in an M2 medium (SIGMA, United States) containing 0.1% hyaluronidase to remove granular cells. The prepared eggs were placed in KSOMaa solution (Caisson Labs, Smithfield, UT, United States) and incubated for at least 8 h in an incubator (37.0°C, 5% CO_2_). The eggs were placed in an incubator for no more than 3 h before injection.

### Preparation of Round Spermatid and Spermatozoa

Sexually mature male C57BL/6 mice were used to obtain round spermatids and spermatozoa. Round spermatids and mature sperm were collected from the testis and epididymis, respectively. Fluorescence-activated cell sorting (FACS) was used to distinguish round spermatids from other cell types. First, part of the seminiferous tubule was broken into pieces and sections using a 1 mL syringe. The suspensions were filtered through a 400-mesh screen (38 μm) and stained with Hoechst 33342 (Beyotime Biotechnology, Haimen, China) at 37.0°C for 10 min. The round spermatids were then isolated using a BD Influx flow cytometer (Becton, Dickinson and Company, Franklin Lakes, NJ, United States).

### ICSI and ROSI

ICSI and ROSI procedures were consistent with previously described methods (Kimura and Yanagimachi, 1995b), with a few differences. We used a PiezoXpert device (Eppendorf, Hamburg, Germany). For ICSI, the inner diameter of the injection needle was approximately 9–10 μm, while for ROSI, it was approximately 6–7 μm. Breaking the zona pellucida and egg membrane require different piezo strengths (intensity = 5 and speed = 15 or intensity = 1 and speed = 1, respectively). For ICSI, the head of the spermatozoa was separated from the tail by applying 1 Piezo pulse (intensity = 5 and speed = 15) to the head-tail junction. The head was subsequently microinjected into an oocyte. For ROSI, eggs need assisted activation before ROSI (see the next section for details). Round spermatids were identified by flow cytometry. Because the inner diameter of the injection needle was small, when a round spermatid was drawn in, the nucleus was separated from the cytoplasm and subsequently microinjected. About ten eggs were injected per 15 min, and the injected oocytes were cultured in balanced KSOMaa solution for at least 8 h in an incubator (37.0°C, 5% CO_2_).

### Oocyte Activation

Because the round spermatids cannot activate the egg by themselves, oocytes were first placed in a Ca^2+^-free CZB medium containing 10 mM SrCl_2_ for 20 min before injection for manual activation.

### Natural *in vivo* Fertilization

Embryos generated using natural *in vivo* fertilization were used as the control group. Male C57BL/6 mice were mated with female B6D2F1 at a ratio of 1:2 in the afternoon. The next morning, the vaginal plug was examined. Mice with a vaginal plug were euthanized, and fertilized eggs were collected and cultured to the 2-cell stage.

### Culture and Observation of Pre-implantation Embryos

Six hours after injection, embryos with a second polar body and male pronucleus formation were classified and further cultured in the ICSI and ROSI groups. The cleavage rate and blastocyst formation conditions were calculated 24 and 96 h after injection, respectively.

### Embryo Transfer

Embryo transfer was performed as previously described (Wang et al., 2020).

### Transcriptome and DNA Methylation Sequencing

Fetuses with pronounced defects were excluded from transcriptome and DNA methylation sequencing analyses. In clinical practice, when fetuses with noticeable defects are identified, the pregnancy is terminated. Eight fetuses and their corresponding placentas identified as morphologically normal were selected for sequencing (No observations of male and female pronuclei formation). Four ICSI-fertilized fetuses and their corresponding placentas served as the ICSI group, and four natural *in vivo* fertilized fetuses and their corresponding placentas served as the control group. Sequencing was performed at Berry Genomics Corporation (Beijing, China).

#### RNA-Sequencing (RNA-seq)

Total RNA was isolated using TRIzol reagent (Invitrogen, Carlsbad, CA, United States). Thirty-two cDNA libraries were constructed, i.e., eight for the ROSI fetuses, eight for the ROSI placentas, eight for the ICSI and control fetuses (four for each group), and eight for the ICSI and control placentas (four for each group). The libraries were sequenced at the Berry Genomics Corporation (Beijing, China) on an Illumina NovaSeq platform, and 150 bp paired-end reads were generated. All clean RNA-sequencing (RNA-seq) data were mapped to the mouse genome (version mm10) using Hisat2 (version 2.1.0) (Kim et al., 2015), and the uniquely mapped reads were used for gene expression level calculations. The gene expression level was normalized by Fragments Per Kilobase per Million (FPKM) using StringTie with -eB parameters (Kovaka et al., 2019). Genes with at least 1 FPKM in at least one sample were used for the next analysis step. Differentially expressed genes (DEGs) were identified by two-fold changes in FPKM between samples and adjusted *P* < 0.05, which were calculated by DEseq2 (Love et al., 2014). For the repeat sequence analysis, clean reads were mapped using the STAR aligner with the–outFilterMultimapNmax 120 parameter (Dobin et al., 2013). TEtranscripts were used to estimate TE abundances based on repeatMasker annotation (Jin et al., 2015). The differentially expressed TEs were also identified by DEseq2 with adjusted *P* < 0.05. The imprinted genes were downloaded from a published paper (Wang et al., 2014). Heatmaps and cluster analysis results were plotted using the heatmap.2 and cluster functions in R., respectively. The violin plots were created using ggplot2.

#### Whole-Genome Bisulfite Sequencing (WGBS)

Approximately 5.2 μg genomic DNA was spiked with 26 ng lambda DNA. The DNA was fragmented to 200–300 bp by sonication with a Covaris S220, followed by end repair and adenylation. Cytosine-methylated barcodes were then ligated to sonicated DNA. The DNA fragments were treated twice with bisulfite using an EZ DNA Methylation-GoldTM Kit. The resulting single-strand DNA fragments were amplified by PCR using KAPA HiFi HotStart Uracil + ReadyMix (2×). A Qubit 2.0 fluorometer was used to quantify the library concentration. Insert size was assessed using an Agilent Bioanalyzer 2100 system. Index-coded samples were clustered with a cBot Cluster Generation System using a TruSeq PE Cluster Kit v3-cBot-HS and sequenced at Berry Genomics Corporation (Beijing, China) on an Illumina Novaseq 6000 platform. 150 bp paired-end reads were generated.

Sequencing adapters and poor-quality reads were removed using Trimmomatic (Bolger et al., 2014). The remaining reads that passed all the filtering steps were counted as clean reads for the next analysis. These clean reads were then mapped into the mouse reference genome (mm10) using BSMAP (Xi and Li, 2009). The parameter (−r 0) was used to filter unique hits. The methylation level for each CpG site was calculated by C/(C + T). The imprinting control regions (ICRs) annotation, WGBS data, and linked GEO datasets for mouse sperm and oocyte samples were downloaded from a published paper (Wang et al., 2014). The methylation distribution among the gene body and differentially methylated regions (DMRs) were plotted with deep-tools (Ramirez et al., 2016). Heatmaps were produced using the heatmap.2 function in R. Violin plots were produced with ggplot2.

### RNA Extraction and qRT-PCR

RNA extraction and qRT-PCR were performed as previously described (Xu et al., 2017). qRT-PCR was performed using an Applied Biosystems QuantStudio 6 Flex (Foster City, CA, United States). Relative gene expression was analyzed using the 2^–ΔΔCt^ method with *GAPDH* as the internal control. All primers are listed in Supplementary Table 1.

### Statistical Analysis

Chi-squared tests were used to compare *in vitro* and *in vivo* developmental efficiency (IBM SPSS 23). Other statistical analyses were performed using GraphPad Prism (GraphPad). Student’s *t*-tests were used for pairwise comparisons of other parameters. Differences were considered significant at *P* < 0.05.

## Results

### Birth Efficiency Was Lower in the ROSI Group Than That in the ICSI Group

Given that ROSI embryos showed diverse pronucleus formation phenotypes, we chose only embryos with male and female pronuclei (2PN) for analyses. In our culture conditions, the cleavage rates of embryos derived upon fertilization with ROSI and ICSI were 100.00% (Supplementary Figure S1A). Unexpectedly, the blastocyst percentage was higher in the ROSI group than that in the ICSI group (88.79% vs. 75.19%, *P* < 0.01) (Supplementary Figure S1B), which may be explained by the smaller diameter of the injection needle used for ROSI (6–7 μm) than for ICSI (9–10 μm). Secondly, as the microdroplets for ICSI–but not ROSI–contain polyvinylpyrrolidone (PVP), a small amount of PVP will inevitably be injected into the cytoplasm during ICSI. The third reason may be that round sperm injection requires pre-oocyte activation, and mature sperm have the ability to activate eggs.

Next, we investigated the *in vivo* development of embryos derived upon using the two methods. Embryos were collected at E11.5, and the survival rate was determined. There was no significant difference in the E11.5 survival rate between the ROSI and ICSI groups (51.61% vs. 55.00%, *P* > 0.05) (Supplementary Figure S1C). The birth rate (i.e., the ability of embryos to develop to term) was lower for ROSI than that for ICSI (22.86% vs. 50.00%, *P* < 0.05) (Supplementary Figure S1D). The developmental data for ROSI- and ICSI-derived embryos are summarized in Table 1.

**TABLE 1:** The development of 2PN embryos derived from intracytoplasmic sperm injection (ICSI) and round spermatid injection (ROSI).

Group	Cleavage rate (%)	Blastocyst rate (%)	E11.5 survival rate (%)	Birth rate (%)
ICSI	100.00 (129/129)	75.19 (97/129)	55.00 (22/40)	50.00 (18/36) *
ROSI	100.00 (116/116)	88.79 (103/116) **	51.61 (16/31)	22.86 (8/35)

2PN, male and female pronuclei; E, embryonic day. *, *P* < 0.05; **, *P* < 0.01.

The developmental efficiency gap of the ROSI and ICSI groups increased from 3.39% at E11.5 (55.00% minus 51.61%) to 27.14% at the developmental maturity stage (50.00% minus 22.86%), indicating that some fetuses were lost after E11.5. This phenomenon prompted us to explore the mechanism underlying the difference in the fetus and placenta between embryos from round spermatids or mature sperm at E11.5. We randomly collected eight E11.5 whole fetuses and corresponding whole placentas from the ROSI group as experimental objects (Supplementary Figure S1E). The crown-rump length (CRL) of the fetuses is 6.23 ± 0.68 cm (6.44, 6.64, 6.36, 7.15, 6.09, 6.55, 5.68, and 4.91, respectively); and the maximum diameter of the placenta is 5.08 ± 0.50 cm (5.56, 4.91, 4.59, 5.70, 5.31, 5.42, 4.85, and 4.29, respectively). All fetuses and placenta have no apparent abnormalities. Four ICSI and *in vivo* fertilized E11.5 fetuses and corresponding placentas were used as control objects (data not shown). Transcriptome and methylation levels of the fetus and placenta were compared among the three groups.

### Transcriptome Analysis of E11.5 Fetuses and Placentas From ROSI

**FIGURE 1:**
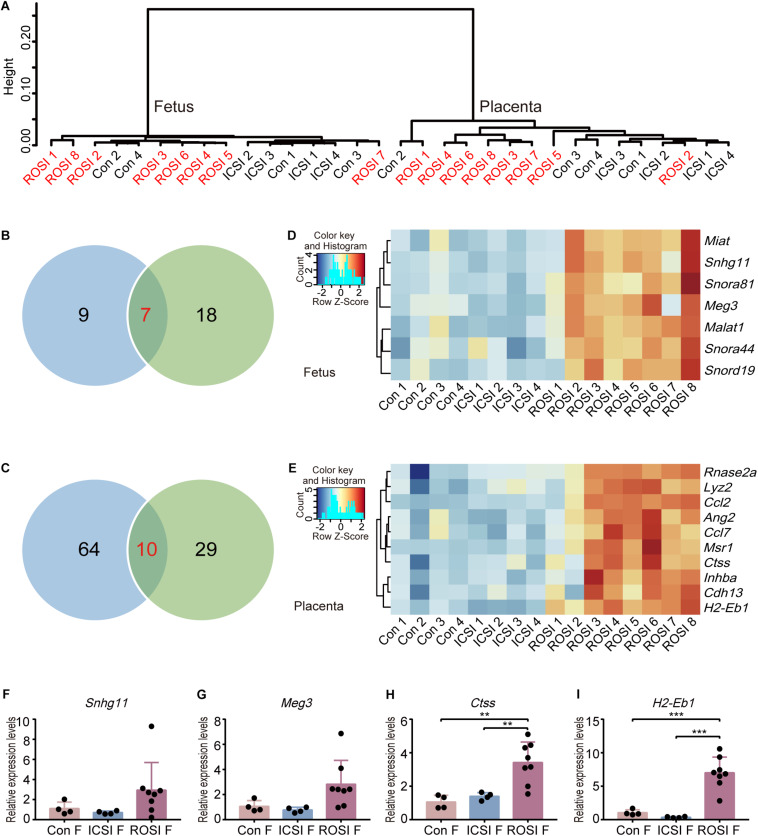
Transcriptome profiles of ROSI-derived fetuses and placentas. **(A)** Unsupervised hierarchical clustering of genes expressed in 16 fetuses and placentas. **(B)** The number of genes expressed differentially between ROSI-derived fetuses and other group fetuses. **(C)** The number of genes expressed differentially between ROSI-derived placentas and other group placentas. **(D)** Heatmap of overlapping differentially expressed genes (DEGs) in fetuses. **(E)** Heatmap of overlapping DEGs in placentas. **(F)** qRT-PCR depicting the expression of *Snhg11* in fetuses. **(G)** qRT-PCR depicting the expression of *Meg3* in fetuses. **(H)** qRT-PCR depicting the expression of *Ctss* in the placenta. **(I)** qRT-PCR depicting the expression of *H2-Eb1* in the placenta. **P < 0.01; ***P < 0.001; Con, Control; F, Fetus; P, Placenta; ROSI, round spermatid injection; ICSI, intracytoplasmic sperm injection.

RNA-sequencing was performed to analyze the fetal and placental transcriptomes. Unsupervised hierarchical clustering analysis was used to compare the transcriptomes of all 16 fetuses and 16 placentas (Figure 1A). As expected, the fetuses and placentas were clearly separated. However, there was no distinct clustering of the fetuses in control, ICSI, and ROSI groups. Similar results were obtained for the 16 placentas, indicating that the overall gene expression patterns were not clearly distinguishable among the three groups. DEGs in pairwise comparisons were further evaluated. In total, nine DEGs were identified between control and ROSI fetuses and 18 DEGs between the ICSI and ROSI fetuses, and seven DEGs were shared in both comparisons (Figure 1B). However, for placentas, 64 DEGs were identified between the control and ROSI groups, 29 DEGs between the ICSI and ROSI groups, and 10 DEGs were shared in the two comparisons (Figure 1C). Heatmap visualization revealed seven and ten overlapping DEGs among the groups in the fetuses (Figure 1D) and placentas (Figure 1E), respectively. Next, low-throughput qRT-PCR was performed for randomly selected DEGs to verify the RNA-seq results (Figures 1F–I). Especially, Maternally expressed gene 3 (*Meg3*), which is located on the murine Dlk1-Dio3 locus, was clearly differentially expressed in ROSI fetuses compared to control or ICSI groups. Seven genes in the Dlk1-Dio3 locus were detected with expression in E11.5 fetuses and placentas. Except for *Meg3*, *Mirg* and *Rian*, which expressed from maternal allele also were up-regulated in ROSI fetuses or placentas but less than 2-fold changes; on the contrary, *Dio3*, which expressed from the paternal allele, was down-regulated in ROSI placentas (Supplementary Figure S2).

**FIGURE 2:**
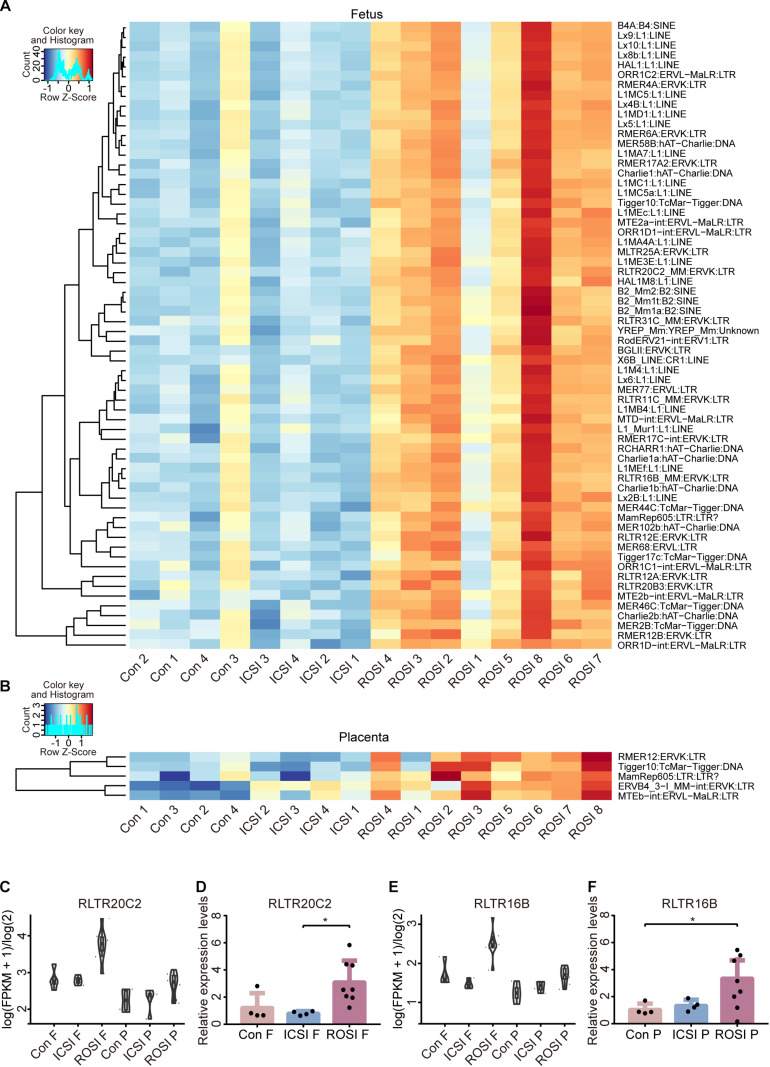
Repetitive DNA sequence expression in ROSI-derived fetuses and placentas. **(A)** Heatmap of RS expression in fetuses. **(B)** Heatmap of RS expression in placentas. **(C)** RNA-seq depicting RS RLTR20C2 expression in fetuses. **(D)** qRT-PCR depicting the expression of RS RLTR20C2 in fetuses. **(E)** RNA-seq depicting RS RLTR16B expression in placentas. **(F)** qRT-PCR depicting the expression of RS RLTR16B in placentas. *, *P* < 0.05; LINEs, long interspersed nuclear elements; SINEs, short interspersed nuclear elements; LTRs, long terminal repeats; Con, Control; F, Fetus; P, Placenta; RS, repetitive DNA sequence.

Repetitive DNA sequences (RS) account for more than 50% of the mammalian genomic DNA (de Koning et al., 2011). However, little is known about their specific functions. The expression of RS in the three groups was assessed, indicating that several RS were differentially expressed in ROSI fetuses (Figure 2A), including 22 long interspersed nuclear elements (LINEs), four short interspersed nuclear elements (SINEs), and 25 long terminal repeats (LTRs). However, only five differentially expressed RS were identified in placentas, most of which were LTRs (Figure 2B). Notably, consistent RS results were obtained by qRT-PCR, thus validating the RNA-seq data (Figures 2C–F).

Given that imprinted genes play an essential role in embryogenesis (Ferguson-Smith and Bourc’his, 2018; Tucci et al., 2019), we performed unsupervised hierarchical clustering analysis of all imprinted genes in the three groups. We did not detect biased clustering, indicating that the expression of imprinted genes did not differ significantly among the fetal (Supplementary Figure S3A) or placental (Supplementary Figure S3B) groups. Next, several important imprinting genes, including *H19* and *Igf2*, were selected for validation by qRT-PCR. The qRT-PCR results were consistent with the RNA-seq data, supporting the reliability of our results (Supplementary Figure S4).

Given that a high abortion rate is a major barrier to practical ROSI implementation (Tanaka et al., 2018), we further analyzed the expression of seven well-studied abortion-related genes in the three groups. *Vegfa*, *Ccl2*, and *Il1b* showed elevated expression in partial fetuses or placentas (Supplementary Figures S5A,B). Moreover, *Ccl2* was highly expressed in the placenta (Supplementary Figure S5C), while *Vegfa* showed high expression in fetuses (Supplementary Figure S5D).

### DNA Methylome of E11.5 Fetuses and Placentas From ROSI

**FIGURE 3:**
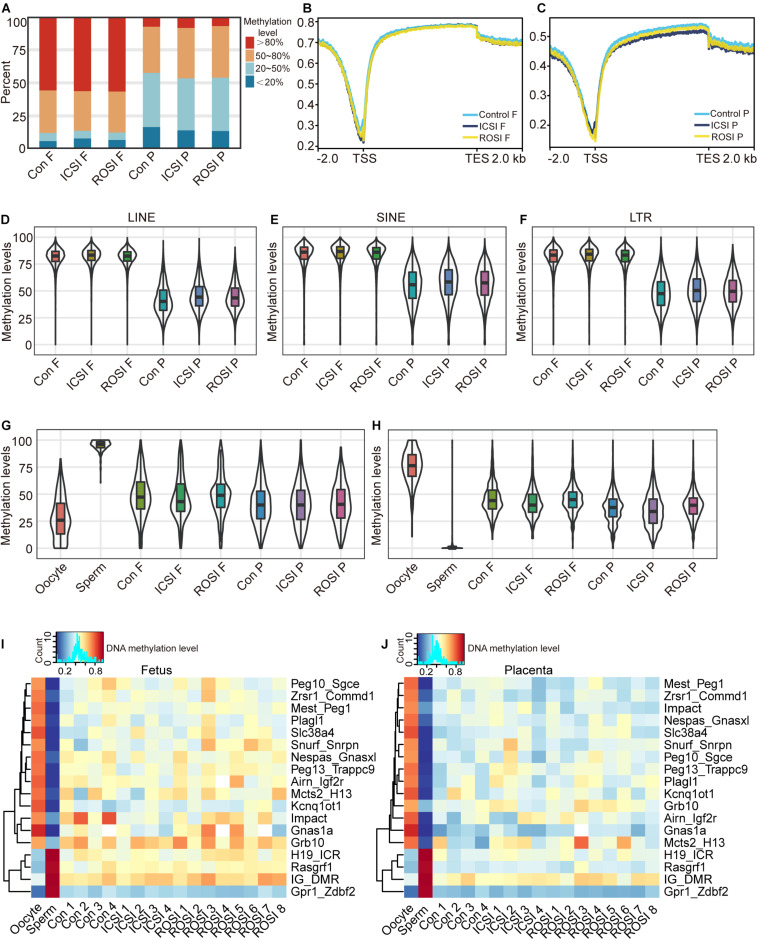
The DNA methylation status of ROSI-derived fetuses and placentas. **(A)** Distribution of genome-wide CpG methylation in different groups of fetuses and placentas. **(B)** CpG methylation at the fetal TSS and TTS. **(C)** CpG methylation at the placental TSS and TTS. Methylation of **(D)** LINEs, **(E)** SINEs, **(F)** LTRs, **(G)** paternally imprinted regions, and **(H)** maternally imprinted regions from fetuses and placentas. Methylation of ICRs from **(I)** fetuses and **(J)** placentas. Con, Control; F, Fetus; P, Placenta; TSS, transcription start site; TTS, transcription termination site; LINEs, long interspersed nuclear elements; SINEs, short interspersed nuclear elements; LTRs, long terminal repeats; ICRs, imprinting control regions.

Given that epigenetic modification, such as DNA methylation, plays a vital role in the regulation of gene expression and disease (Greenberg and Bourc’his, 2019), we next evaluated the fetal and placental DNA methylation status. WGBS was used for DNA methylation level detection. The whole genome DNA methylation levels were evaluated with CpG sites covered by at least 10 reads. Notably, the DNA methylation status of all CpG sites were similar in the fetuses and placentas of all three groups (Figure 3A). In particular, more than 50% of the CpG sites in the fetuses were hypermethylated (>80%), while most of the CpG sites in the placentas showed intermediate methylation levels (20–80%) (Figure 3A). Furthermore, we found that in both fetuses and placentas, transcription start site (TSS) regions were hypomethylated, and transcription termination site (TTS) regions were hypermethylated (Figures 3B,C). Methylation patterns of the promoter regions of DEGs between ROSI and control or ICSI groups also were not detected with significant changes (Supplementary Figure S6). As described above, RS showed differential expression profiles among the three groups, which prompted us to assess whether this could be explained by a difference in the DNA methylation status. We divided RS into different types according to RepeatMasker annotation. Notably, we observed similar methylation patterns in LINEs (Figure 3D), SINEs (Figure 3E), and LTRs (Figure 3F) among the three groups. Further, RS elements were hypermethylated in the fetuses and hypomethylated in the placentas, suggesting that DNA methylation and other factors may act together to regulate RS expression.

Next, the DNA methylation levels of the paternal and maternal ICRs in sperm and oocytes were analyzed. Only confirmed ICRs in a previous publication (Wang et al., 2014) were used for the analysis. Our results revealed similar methylation profiles between the three groups (Figures 3G,H). The expression of imprinted genes is regulated through the methylation of ICRs, and most ICRs are hypermethylated in oocytes and hypomethylated in sperm (Smallwood et al., 2011). In line with the finding that there were no significant differences in the expression of imprinted genes between the three groups, we observed that the ICRs of the fetuses and placentas from the three groups were similarly methylated (Figures 3I,J). Especially, the *Meg3* gene located in the Dlk1-Dio3 region was differentially expressed in RNA levels. In contrast, the Dlk1-Dio3 intergenic differentially methylated region (IG-DMR), which regulates the gene expression pattern of the whole region, did not show the different methylation status between the ICSI and ROSI fetuses. Maybe the factors beyond DNA methylation regulated the expression of *Meg3*.

The *de novo* identification of DMR was performed by metilene (Juhling et al., 2016), CpG site with more than 10% methylation difference between ICSI and ROSI groups were labeled as differentially methylated CpG site (DMC). More than five continuous labeled DMCs while with less than 500 bp maximum distance between 2 DMCs were recorded as one DMR. In fetal tissues, 119 hypermethylated DMRs and 272 hypomethylated DMRs were identified in the ROSI group compared with the ICSI group (Supplementary Figure S7A). In placental tissues, 665 hypermethylated DMRs and 1519 hypomethylated DMRs were identified in the ROSI group compared with the ICSI group (Supplementary Figure S7B). Checking the expression levels of genes overlapped with these DMRs and we did not detect consistently differentially expressed patterns with DMRs (Supplementary Figures S7C–F).

### Transcriptomes and DNA Methylomes of E11.5 ROSI-Derived Fetuses and Placentas With Different Crown-Rump Lengths

**FIGURE 4:**
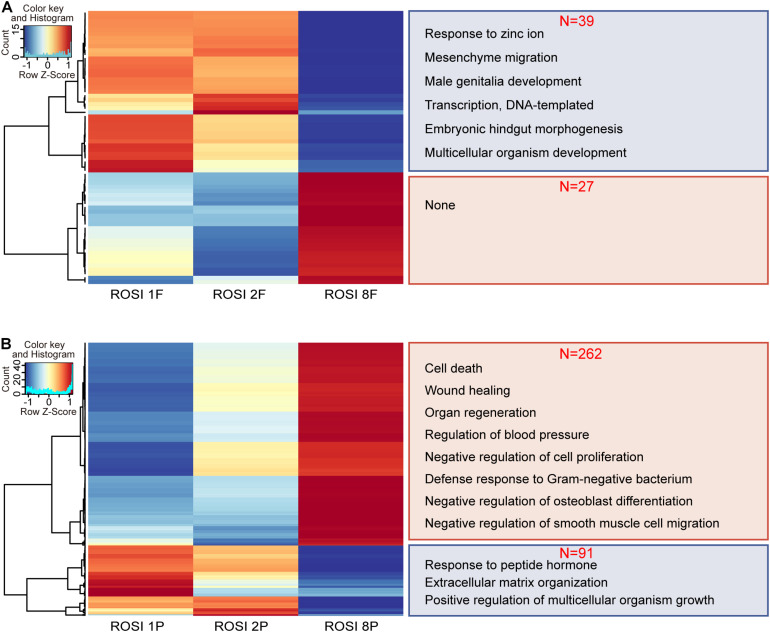
The transcriptome of ROSI-derived fetuses and placentas with abnormal crown-rump length. **(A)** Heatmap of genes differentially expressed between fetuses of normal CRL (ROSI 1 and ROSI 2) and abnormal CRL (ROSI 8). Enriched GO terms (biological process) associated with down-regulated genes in the abnormal CRL sample are shown on the right (39 vs. 27). **(B)** Heatmap of genes differentially expressed between placentas of normal CRL and abnormal CRL. Enriched GO terms (biological process) associated with down- or up-regulated genes in the abnormal CRL sample are shown on the right (262 vs. 91). Con, Control; F, Fetus; P, Placenta; CRL, Crown-rump length; GO, Gene Ontology.

Given the similarities in the transcriptome and DNA methylation levels among the three groups, we investigated the differences between fetuses and placentas from ROSI. The CRL is a common indicator of fetal quality. Accordingly, we selected fetus no. 8 as an abnormally developed embryo because its CRL was less than 6 mm (the standard size at this period). Fetuses no. 1 and no. 2 with CRLs greater than 6 mm were selected as controls. Thirty-nine genes were down-regulated and twenty-seven genes were up-regulated in fetus no. 8 compared with fetuses no. 1 and no. 2. The down-regulated genes were enriched for functions in embryonic hindgut morphogenesis (Figure 4A). However, in the placental tissues of fetus no. 8, we observed 262 up-regulated genes, which were mainly involved in pathways related to vasoconstriction, cell growth regulation, and vasculogenesis (Figure 4B). The 91 down-regulated genes were primarily enriched for functions in the extracellular matrix organization, estradiol response, and fatty acid transport (Figure 4B). Moreover, 14 differentially expressed RS, including one LINE, two SINEs, and eight LTRs, were identified in fetus no. 8 (Supplementary Figure S8A). However, in placenta no. 8, only four RS, including one LINE and three LTRs, were differentially expressed (Supplementary Figure S8B). With respect to DMRs, with more stringent filtering that 20% methylation difference and 10 continuous DMCs in DMR definition by metilene, we identified 234 hypermethylated and 69 hypomethylated DMRs in fetus no. 8 (Supplementary Figure S9A). In addition, we identified 2185 hypermethylated DMRs and 203 hypomethylated DMRs in the placenta of fetus no. 8 (Supplementary Figure S9B).

**FIGURE 5:**
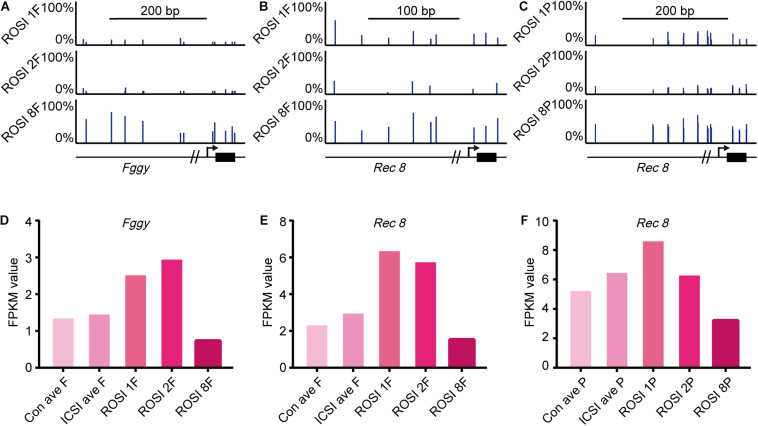
Correlation between gene expression and DNA methylation in ROSI-derived fetuses and placentas. **(A)** Representative CpG methylation profile of the *Fggy* promoter in normal and abnormal CRL fetuses. **(B)** Representative CpG methylation profile of the *Rec8* promoter in normal and abnormal CRL fetuses. **(C)** Representative CpG methylation profile of the *Rec8* promoter in normal and abnormal CRL placentas. **(D)** Fragments Per Kilobase per Million (FPKM) values for *Fggy* in normal and abnormal CRL fetuses. **(E)** FPKM values for *Rec8* in normal and abnormal CRL fetuses. **(F)** FPKM values for *Rec8* in normal and abnormal CRL placentas. Con, Control; F, Fetus; P, Placenta; CRL, Crown-rump length.

We could not identify a relationship between gene expression and DNA methylation in the overall ROSI group. However, in fetus no. 8 and controls (CRL > 6 mm) in the ROSI group, we found a relationship between the methylation status of promoter regions and the expression of *Fggy* (Figure 5A) and *Rec8* (Figure 5B). Additionally, a relationship was identified between the methylation status of promoter regions and the expression of *Rec8* (Figure 5C) in placenta no. 8. The promoter regions of these genes were hypermethylated in fetus and placenta no. 8, and accordingly, the mRNA expression of these genes was reduced (Figures 5D–F).

## Discussion

Although ROSI technology is highly promising for patients with non-obstructive azoospermia, inefficiency and safety concerns have hampered its clinical application on a broader scale (ASRM and SART, 2006). Moreover, the artificial fertilization method used in ROSI and ICSI may lead to abnormal reprogramming of the paternal genome and further developmental abnormalities in the embryos (Yanagimachi, 2005). Recently, owing to the development of various high-throughput sequencing technologies, our understanding of epigenetic modifications during embryonic development has improved substantially (Guo et al., 2015; Wu et al., 2018; He P. et al., 2020; He Y. et al., 2020). Given that the placenta is essential for establishing and maintaining a healthy pregnancy and regulating fetal growth as well as maternal adaptation to pregnancy (Maltepe and Fisher, 2015), we analyzed the transcriptome and DNA methylation profiles in the fetus and placenta. Our characterization of the transcriptome and DNA methylome of ROSI-derived E11.5 fetuses and placentas could provide an experimental basis for evaluating the safety of this technology.

Although the whole transcriptome did not show a clear difference among samples generated by three different fertilization methods, we have identified several DEGs, including seven overlapping DEGs in fetuses in both comparisons (ROSI vs. control and ROSI vs. ICSI) and ten overlapping DEGs in placentas. *Meg3* was also an overlapping DEG in fetuses. *Meg3* is a long non-coding RNA expressed in many tissues and is involved in many biological processes, such as development, metastasis, and metabolic processes in cells (Mondal et al., 2015; Ghafouri-Fard and Taheri, 2019). Our results indicated that *Meg3* expression is higher in ROSI fetuses than that in control or ICSI fetuses. *Meg3* is located on the murine Dlk1-Dio3 locus. In cloned embryos, the loss of Dlk1-Dio3 imprinting is observed in nearly half of the cloned embryos and is highly correlated with embryonic lethality (Okae et al., 2014). *Meg3* expression abnormalities observed in ROSI fetuses may also be associated with late embryo loss during pregnancy. Cathepsin S (*Ctss*), an overlapping DEG in the placenta, encodes a member of the peptidase C1 (papain) family of cysteine proteases. *Ctss* homozygous knockout mice exhibit impaired wound healing, reduced tumorigenesis in a pancreatic cancer model, and reduced pathogenesis in a myasthenia gravis model (Tjondrokoesoemo et al., 2016). Our results indicate that *Ctss* expression was higher in ROSI placentas than that in control or ICSI placentas, and that this high expression may negatively affect placental development. The effects of other DEGs, including *Miat*, *Snhg11*, *Rnase2a*, and *Ang2*, on fetal and placental development, require further study.

Epigenetic modification refers to the reversible and hereditary alteration of gene function in the absence of nuclear DNA sequence changes, including DNA methylation, histone modification, chromatin accessibility, and chromatin structural changes (Bird, 2007; Berger et al., 2009). DNA methylation refers to the process by which a methyl group is added to DNA molecules by DNA methyltransferase (DNMT). In mammals, DNA methylation usually occurs at the fifth carbon atom of cytosine in CpG dinucleotides (Holliday and Pugh, 1975). During mammalian gametogenesis and early embryonic development, DNA methylation results in overall reprogramming. In mice, two genome-wide DNA methylation removal and remodeling processes exist; one occurs from fertilization to germ layer differentiation (Saitou et al., 2012; Wu and Zhang, 2014), and the other occurs during gametogenesis. However, embryonic and placental tissue undergo different methylation remodeling processes. Indeed, the methylation levels in placental tissue are generally lower than those in embryonic tissues (Smith et al., 2017). Consistent with these findings, methylation levels of E11.5 ROSI-derived fetuses were higher than those of placentas. Active demethylation in the male pronucleus of ROSI embryos is abnormal (Kishigami et al., 2006; Kurotaki et al., 2015) and leads to abnormal embryonic development. Although some DMRs were identified, there are no significant changes in whole genome-wide methylation patterns in E11.5 fetuses and placentas among the three groups, especially in ICRs. These results indicated that the main methylation pattern across all CpG sites in E11.5 ROSI fetuses and placentas are normal.

Imprinting disorders are caused by genetic defects or epigenetic mutations (DNA methylation), such as aberrant DNA methylation of DMRs involved in regulating the allele-specific expression of imprinted genes (Butler, 2009). Except for DNA methylation could regulate imprinted genes, some other histone methylations such as H3K27me3 could regulate the expression of allele-specific genes through non-canonical imprinting mode (Inoue et al., 2017a,b). We predicted that some modifications beyond DNA methylation might participate in regulating imprinted gene expression like *Meg3* in the Dlk1-Dio3 region. Many studies have demonstrated that ART might be related to rare imprinting disorders, such as Angelman syndrome, Beckwith–Wiedemann syndrome, Silver–Russell syndrome, and Prader–Willi syndrome (Lucifero et al., 2004; Chiba et al., 2013; Matsubara et al., 2016; Mussa et al., 2017; Cortessis et al., 2018; Uk et al., 2018). ART may prevent the proper erasure, establishment, and maintenance of DNA methylation. A nationwide epidemiological study in Japan in 2015 revealed a 4.46- and 8.91-fold increase in frequencies of Beckwith–Wiedemann syndrome and Silver–Russell syndrome in children conceived using ART compared to in the general population (Hattori et al., 2019). However, some studies did not identify a relationship between ART and imprinting disorders (Lidegaard et al., 2005; Doornbos et al., 2007). For ROSI–other than potential imprinting defects caused by the ART technology itself–whether the imprinting pattern of round spermatids differs from that of mature sperm is unclear. In our study, no significant difference was observed in the expression of more than 100 imprinted genes in fetuses and placentas at E11.5 among the three groups. ICR methylation regulates the expression of imprinted genes. Our study showed that the methylation levels of 17 ICRs were not abnormal in the ROSI group, consistent with the normal expression level of imprinted genes.

RS refers to a class of DNA sequences that can move to different genomic locations. These low-complexity elements (e.g., LINEs and SINEs) account for up to two-thirds of the human genome (de Koning et al., 2011). Despite their abundance, repetitive DNAs are often ignored in genomic studies owing to technical challenges in identification, assembly, and quantification. In ROSI fetuses, we found many differentially expressed RS. Further studies are needed to determine whether abnormally activated RS is associated with high abortion rates in ROSI fetuses. However, RS methylation levels were similar among groups, indicating that other mechanisms may regulate RS expression.

The abortion rate of ROSI embryos is very high (Tanaka et al., 2018). We found that the E11.5 survival rates were similar in the ROSI and ICSI groups, but the gap in maturation efficiency was higher in the ROSI group. Moreover, as we terminated the pregnancy halfway, we could not evaluate subsequent developmental processes. We artificially categorized ROSI fetuses as normal or abnormal according to the CRL and the corresponding increased risk of late pregnancy loss for fetuses with a CRL less than 6 mm (e.g., fetus no. 8) due to the abnormal expression of abortion-related genes. We found a correlation between the abnormal hypermethylation of two genes and decreased gene expression. In both the fetus and placenta, *Rec8* was hypermethylated in the promoter region, and mRNA expression was reduced in the abnormal CRL group. Rec8 is crucial for diverse aspects of chromosome dynamics during meiosis, especially in meiotic chromosome organization and recombination (Bardhan, 2010). *Rec8* homozygous null mice are infertile and exhibit small ovaries and testes. Abnormal *Rec8* hypermethylation and expression in fetus and placenta no. 8 may adversely affect future fetal development or the reproductive ability of the offspring. Abnormal hypermethylation may be associated with the low open chromatin state of round spermatids during the reprogramming process.

Despite considerable efforts to evaluate different developmental data types in ROSI-derived fetuses and placentas, it is essential to note that our study had some limitations. We did not separate the tissues or organs of fetuses from ROSI for more precise analyses. How other epigenetic modifications, such as histone modifications, affect fetal gene expression should be further evaluated. In addition, we did not study gene expression or DNA methylation patterns at different developmental stages, which is crucial for understanding the whole process of fetal development. These points each warrant further detailed investigations to provide a theoretical basis for the safe and efficient application of ROSI in clinical settings.

## Conclusion

In conclusion, our results showed that round spermatid fertilization and mature spermatozoa fertilization yield similar overall transcriptome profiles in E11.5 fetuses and placentas. The general methylation levels of fetuses and placentas derived upon fertilization using round spermatids or mature sperm were similar. Furthermore, there were no differences in ICR methylation patterns or the expression of most imprinted genes. However, many repetitive DNA sequences were abnormally activated in ROSI-derived E11.5 fetuses. Two genes associated with hypermethylated promoter regions were aberrantly expressed in a ROSI fetus with an abnormal CRL. These results improve our understanding of the molecular determinants of the inefficiency of the ROSI technique and provide a basis for enhancing the safe and efficient application of this method.

## Data Availability Statement

The raw sequence data reported in this paper have been deposited in the Genome Sequence Archive (Wang et al., 2017) in National Genomics Data Center (CNCB-NGDC Members and Partners, 2021), China National Center for Bioinformation/Beijing Institute of Genomics, Chinese Academy of Sciences, under accession number CRA003905 that are publicly accessible at https://bigd.big.ac.cn/gsa.

## Ethics Statement

The animal study was reviewed and approved by Experimental Animal Ethics Committee of the First Hospital of Jilin University.

## Author Contributions

HZ and GF participated in the data analysis and drafted the manuscript. DY and TL conceived the study. RL, ZL, CL, XD, and YZ assisted with experimental design. HZ, HS, and YC conducted the experiments. WL, QZ, and TH obtained the funding. All authors contributed significantly, and that all authors are in agreement with the content of the manuscript.

## Conflict of Interest

The authors declare that the research was conducted in the absence of any commercial or financial relationships that could be construed as a potential conflict of interest.

## Supplementary Material

Supplementary Figure 1 Developmental efficiency and representative images of fetuses and placentas derived from round spermatid injection. **(A)** Cleavage rate of 2PN source embryos derived from ICSI and ROSI (2PN embryos, female and male pronuclei). **(B)** Blastocyst rate of 2PN source embryos derived from ICSI and ROSI. **(C)** The survival rate of embryonic day **(E)** 11.5 embryos originating from 2PN source embryos derived from ICSI and ROSI. **(D)** The birth rate associated with 2PN source embryos derived from ICSI and ROSI. **(E)** Eight randomly selected E11.5 fetuses and corresponding placentas derived from ROSI, four natural *in vivo* fertilization fetuses and corresponding placentas (control group; data not shown), four fetuses, and corresponding placentas derived from ICSI (ICSI group; data not shown). Scale bar, 1 mm; S, survival; ROSI, round spermatid injection; ICSI, intracytoplasmic sperm injection; ^∗^*P* < 0.05; ^∗∗^*P* < 0.01.

Supplementary Figure 2 Expression levels of genes located in the Dlk1-Dio3 region. **(A)** Expression levels of *Meg3*. **(B)** Expression levels of *Dio3*. **(C)** Expression levels of *Dio3os*. **(D)** Expression levels of *Dlk1*. **(E)** Expression levels of *Mirg*. **(F)** Expression levels of *Rian*. **(G)** Expression levels of *Rtl1*. Con, Control; F, Fetus; P, Placenta; ^∗^*P* < 0.05; ^∗∗^*P* < 0.01.

Supplementary Figure 3 Heatmap of imprinted genes in ROSI-derived fetuses and placentas. **(A)** Heatmap depicting the expression of imprinted genes in fetuses. **(B)** Heatmap depicting the expression of imprinted genes in the placenta.

Supplementary Figure 4 qRT-PCR depicting the expression of some imprinted genes in ROSI-derived fetuses and placentas. **(A)** *H19* expression in the fetuses. **(B)** *H19* expression in the placentas. **(C)** *Igf2* expression in the fetuses. **(D)** *Igf2* expression in the placentas. **(E)** *Peg10* expression in the fetuses. **(F)** *Peg10* expression in the placentas. **(G)** *Ascl2* expression in the fetuses. **(H)** *Ascl2* expression in the placentas. *P* > 0.05. Con, Control; F, Fetus; P, Placenta.

Supplementary Figure 5 Expression of abortion-related genes in ROSI-derived fetuses and placentas. **(A)** Heatmap depicting the expression of seven abortion-related genes in fetuses. **(B)** Heatmap depicting the expression of seven abortion-related genes in placentas. **(C)** RNA-seq results for *Ccl2* expression. **(D)** RNA-seq results for *Vegfa* expression.

Supplementary Figure 6 DNA methylation level analysis for the promoter regions of differentially expressed genes (DEGs) between ROSI samples and other groups. **(A)** DNA methylation level analysis for the promoter regions of DEGs between control and ROSI fetuses. **(B)** DNA methylation level analysis for the promoter regions of DEGs between ICSI and ROSI fetuses. **(C)** DNA methylation level analysis for the promoter regions of DEGs between control and ROSI placentas. **(D)** DNA methylation level analysis for the promoter regions of DEGs between ICSI and ROSI placentas.

Supplementary Figure 7 Methylation status of regions methylated differentially between ROSI and ICSI fetuses and placentas and the expression level of genes within the identified DMRs. **(A)** DNA methylation status of differentially methylated regions (DMRs) between ROSI and ICSI fetuses. **(B)** DNA methylation status of DMRs between ROSI and ICSI placentas. **(C)** The expression level of genes located in the hypomethylated DMRs in the ROSI fetal tissue. **(D)** The expression level of genes located in the hypermethylated DMRs in the ROSI fetal tissue. **(E)** The expression level of genes located in the hypomethylated DMRs in the ROSI placental tissue. **(F)** The expression level of genes located in the hypermethylated DMRs in the ROSI placental tissue.

Supplementary Figure 8 Repetitive sequence (RS) expression in ROSI-derived fetuses and placentas with abnormal crown-rump length. **(A)** Heatmap depicting RS expression in normal and abnormal crown-rump length (CRL) fetuses. **(B)** Heatmap depicting RS expression in normal and abnormal CRL placentas.

Supplementary Figure 9 DNA methylation status of DMRs between fetuses and placentas with normal and abnormal CRL. **(A)** DNA methylation status of DMRs between normal and abnormal CRL fetuses. **(B)** DNA methylation status of DMRs between normal and abnormal CRL placentas.
