# Supplementary figures and images for: Transcriptome and DNA Methylation Profiles of Mouse Fetus and Placenta Generated by Round Spermatid Injection
Source: Front Cell Dev Biol. 2021 Mar 16;9:632183. doi: 10.3389/fcell.2021.632183 (PMC8009284; doi:10.3389/fcell.2021.632183)

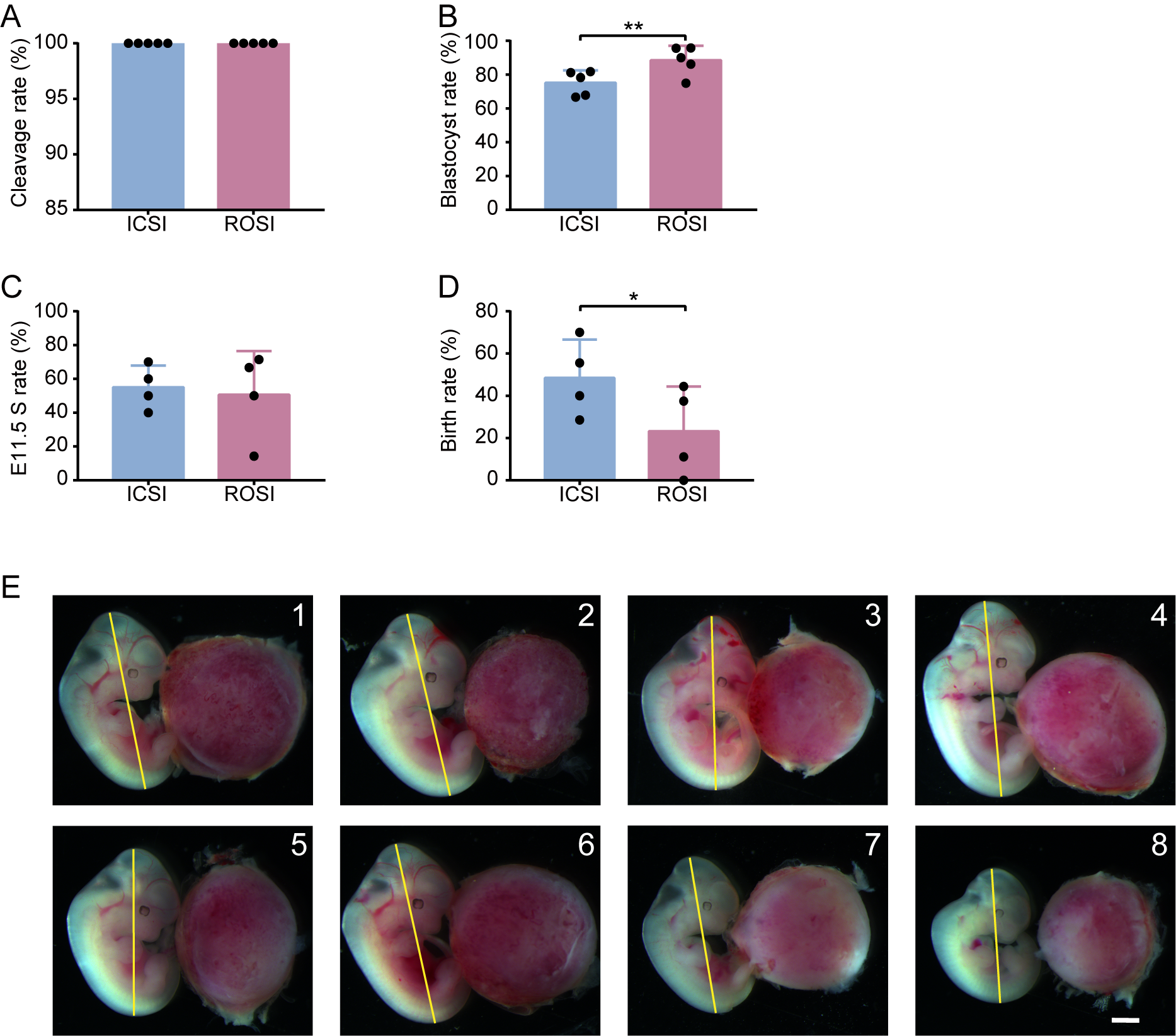

Supplement: Supplementary Figure 1 — Developmental efficiency and representative images of fetuses and placentas derived from round spermatid injection. (A) Cleavage rate of 2PN source embryos derived from ICSI and ROSI (2PN embryos, female and male pronuclei). (B) Blastocyst rate of 2PN source embryos derived from ICSI and ROSI. (C) The survival rate of embryonic day (E) 11.5 embryos originating from 2PN source embryos derived from ICSI and ROSI. (D) The birth rate associated with 2PN source embryos derived from ICSI and ROSI. (E) Eight randomly selected E11.5 fetuses and corresponding placentas derived from ROSI, four natural in vivo fertilization fetuses and corresponding placentas (control group; data not shown), four fetuses, and corresponding placentas derived from ICSI (ICSI group; data not shown). Scale bar, 1 mm; S, survival; ROSI, round spermatid injection; ICSI, intracytoplasmic sperm injection; ∗P < 0.05; ∗∗P < 0.01. [file Image_1.tif]

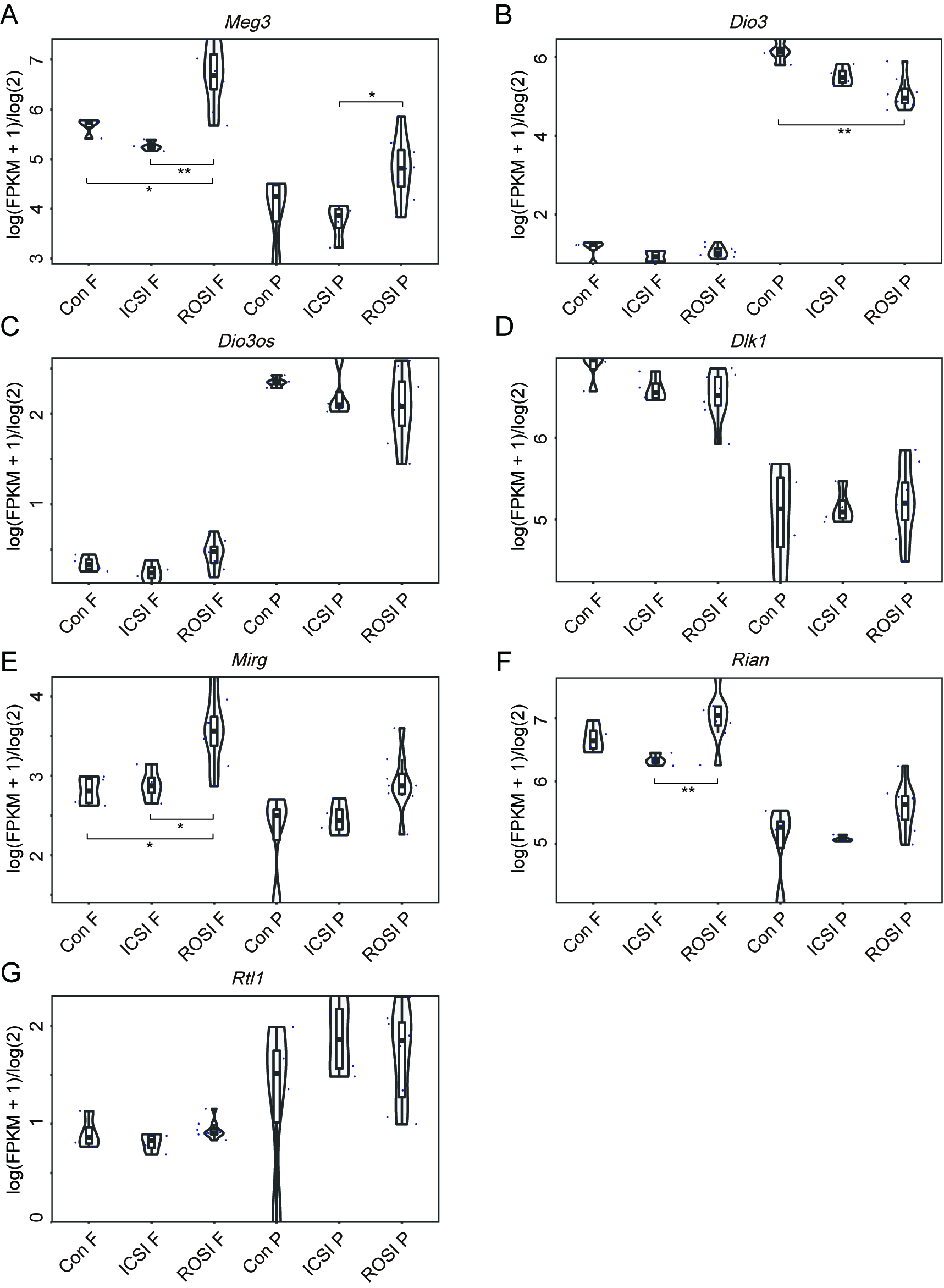

Supplement: Supplementary Figure 2 — Expression levels of genes located in the Dlk1-Dio3 region. (A) Expression levels of Meg3. (B) Expression levels of Dio3. (C) Expression levels of Dio3os. (D) Expression levels of Dlk1. (E) Expression levels of Mirg. (F) Expression levels of Rian. (G) Expression levels of Rtl1. Con, Control; F, Fetus; P, Placenta; ∗P < 0.05; ∗∗P < 0.01. [file Image_2.tif]

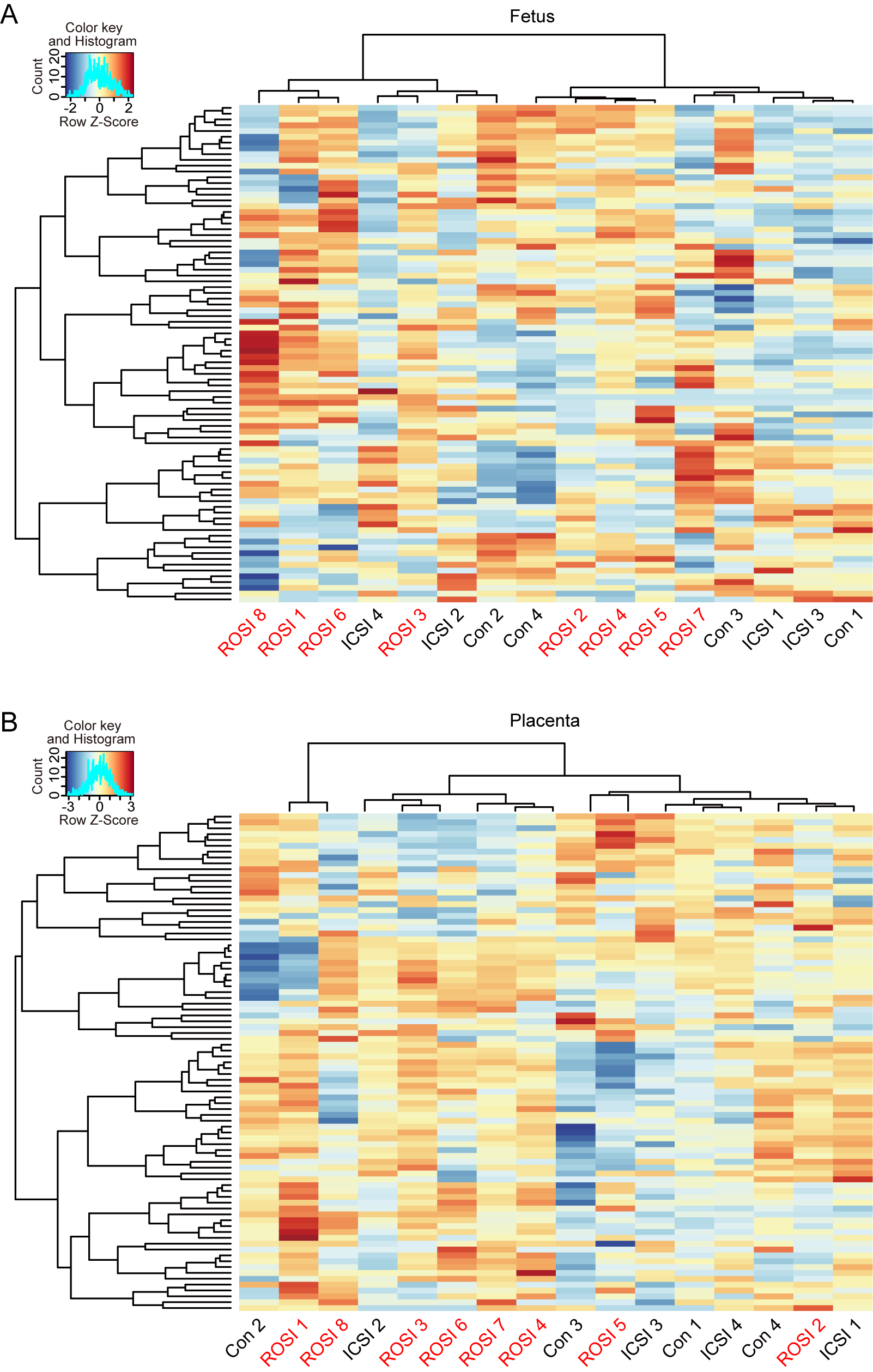

Supplement: Supplementary Figure 3 — Heatmap of imprinted genes in ROSI-derived fetuses and placentas. (A) Heatmap depicting the expression of imprinted genes in fetuses. (B) Heatmap depicting the expression of imprinted genes in the placenta. [file Image_3.tif]

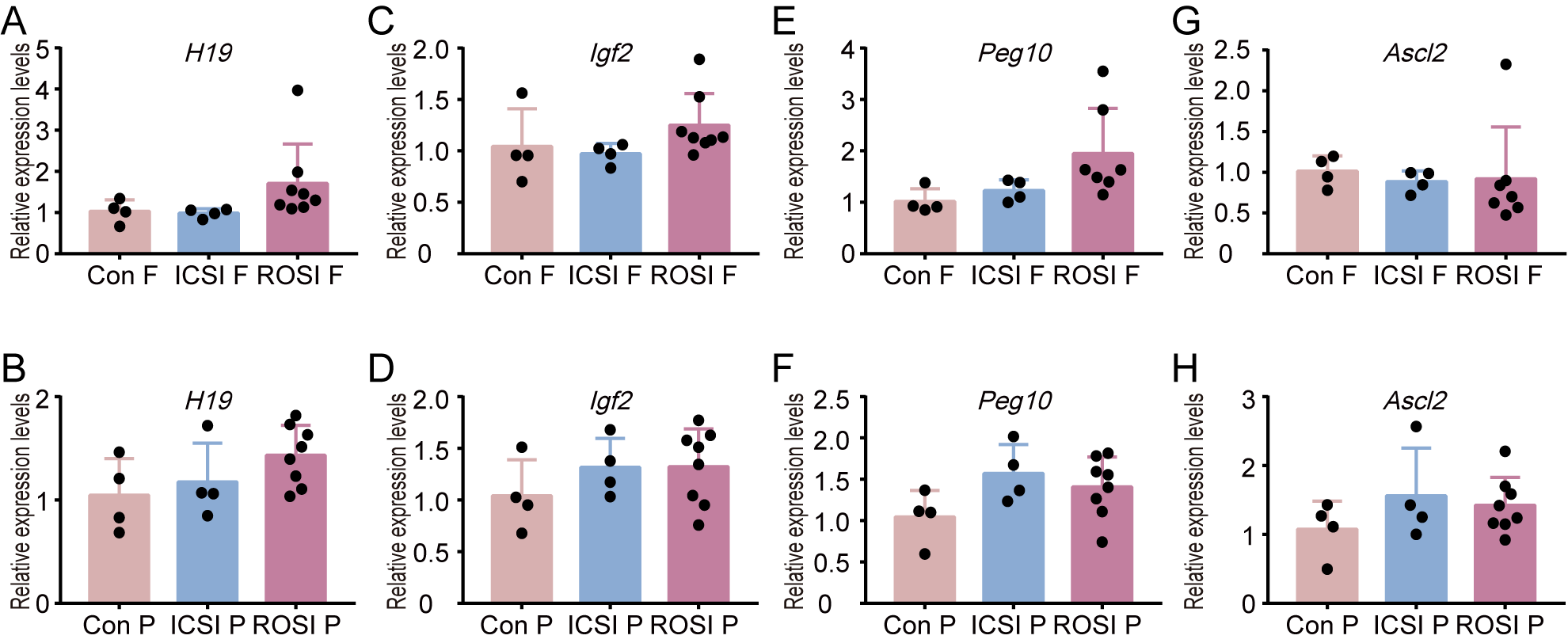

Supplement: Supplementary Figure 4 — qRT-PCR depicting the expression of some imprinted genes in ROSI-derived fetuses and placentas. (A) H19 expression in the fetuses. (B) H19 expression in the placentas. (C) Igf2 expression in the fetuses. (D) Igf2 expression in the placentas. (E) Peg10 expression in the fetuses. (F) Peg10 expression in the placentas. (G) Ascl2 expression in the fetuses. (H) Ascl2 expression in the placentas. P > 0.05. Con, Control; F, Fetus; P, Placenta. [file Image_4.tif]

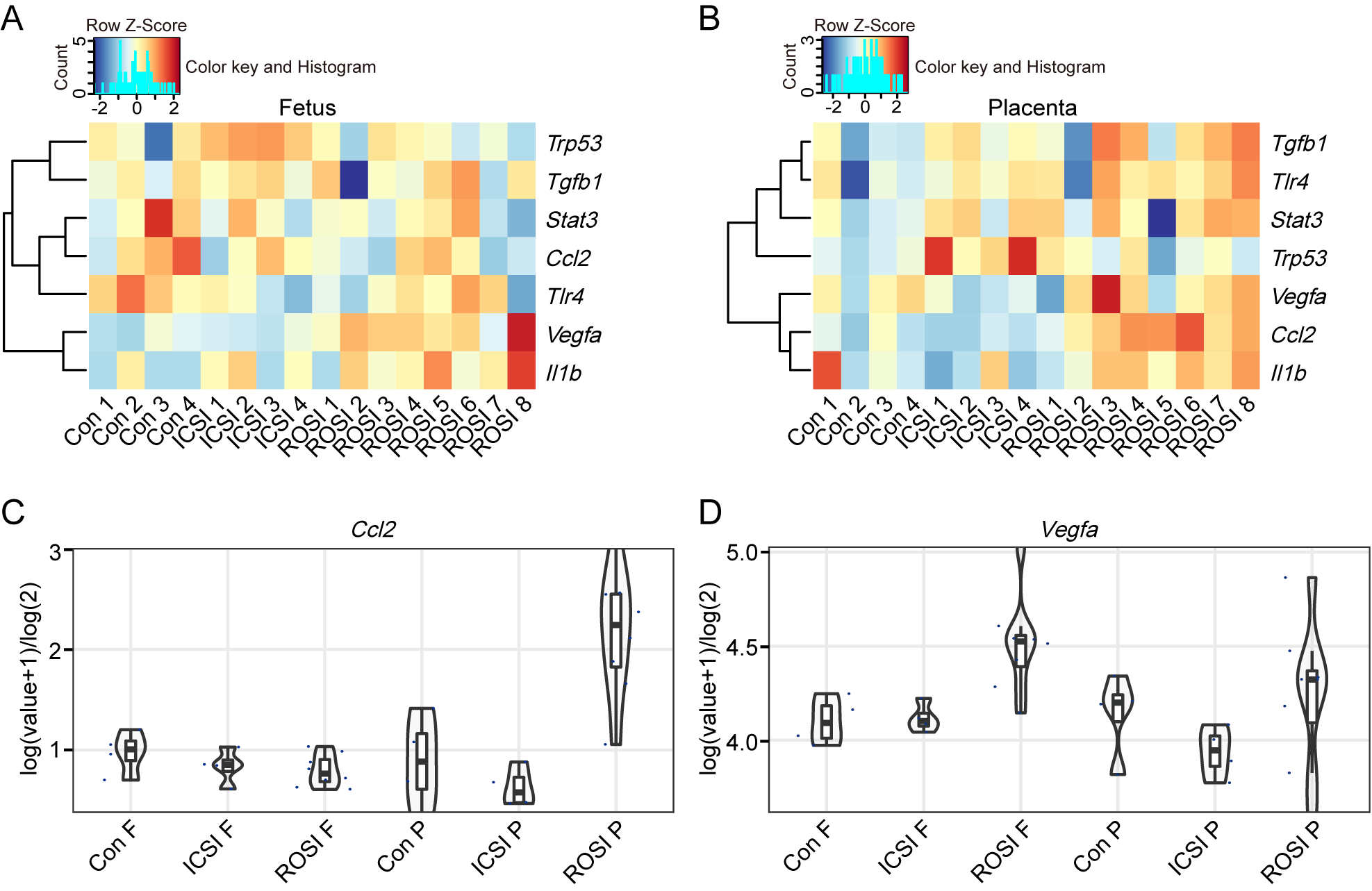

Supplement: Supplementary Figure 5 — Expression of abortion-related genes in ROSI-derived fetuses and placentas. (A) Heatmap depicting the expression of seven abortion-related genes in fetuses. (B) Heatmap depicting the expression of seven abortion-related genes in placentas. (C) RNA-seq results for Ccl2 expression. (D) RNA-seq results for Vegfa expression. [file Image_5.tif]

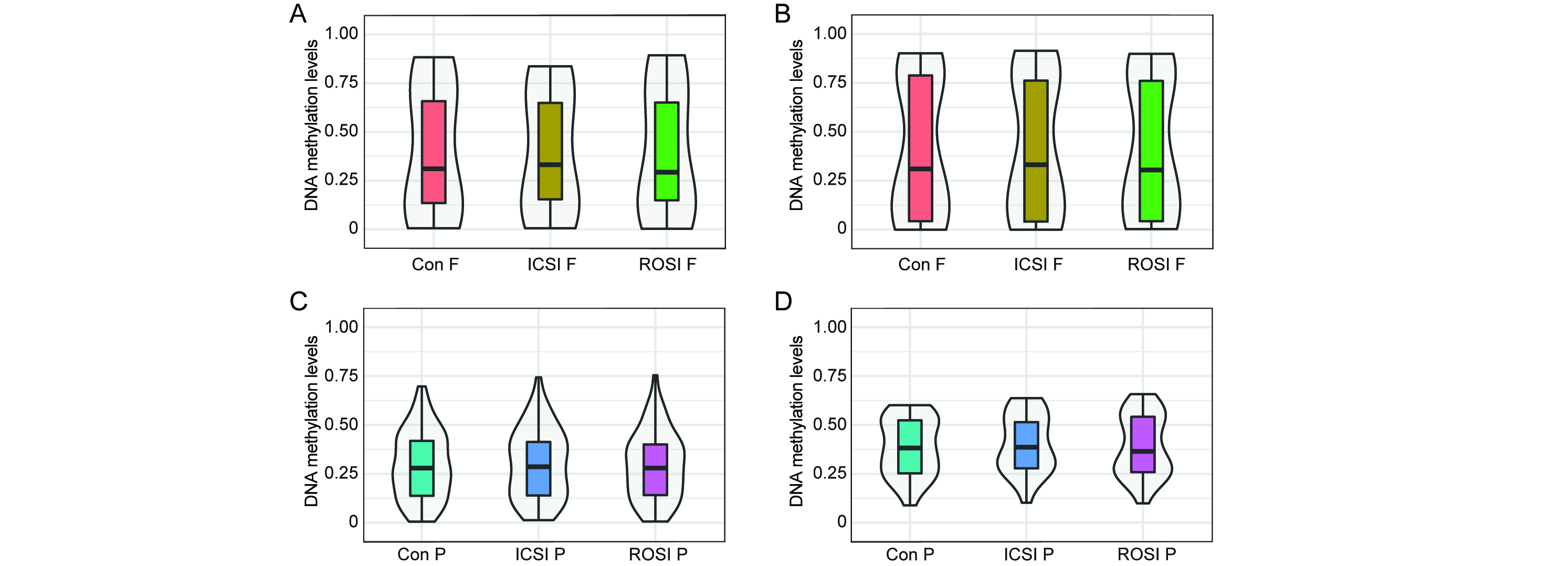

Supplement: Supplementary Figure 6 — DNA methylation level analysis for the promoter regions of differentially expressed genes (DEGs) between ROSI samples and other groups. (A) DNA methylation level analysis for the promoter regions of DEGs between control and ROSI fetuses. (B) DNA methylation level analysis for the promoter regions of DEGs between ICSI and ROSI fetuses. (C) DNA methylation level analysis for the promoter regions of DEGs between control and ROSI placentas. (D) DNA methylation level analysis for the promoter regions of DEGs between ICSI and ROSI placentas. [file Image_6.tif]

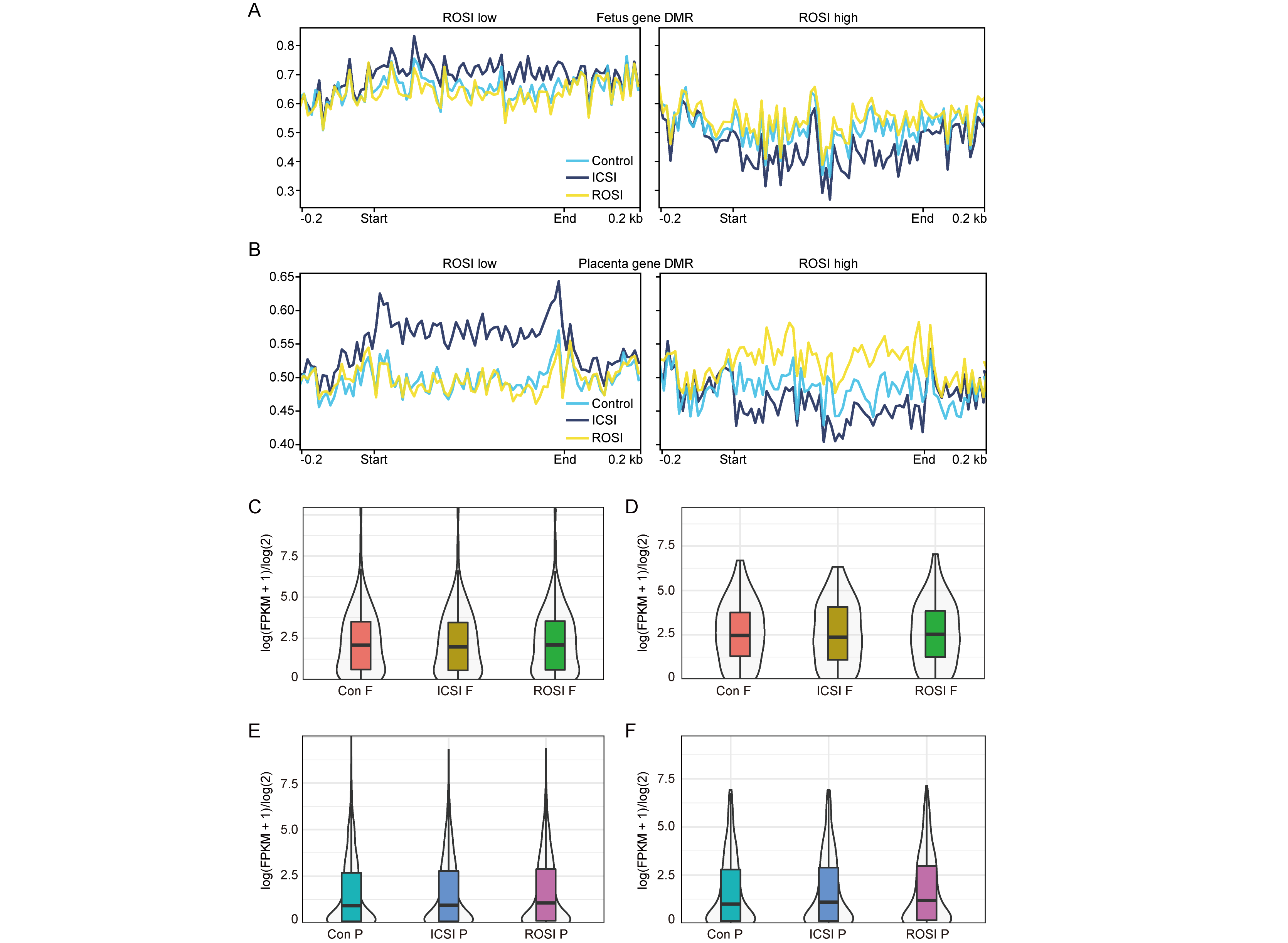

Supplement: Supplementary Figure 7 — Methylation status of regions methylated differentially between ROSI and ICSI fetuses and placentas and the expression level of genes within the identified DMRs. (A) DNA methylation status of differentially methylated regions (DMRs) between ROSI and ICSI fetuses. (B) DNA methylation status of DMRs between ROSI and ICSI placentas. (C) The expression level of genes located in the hypomethylated DMRs in the ROSI fetal tissue. (D) The expression level of genes located in the hypermethylated DMRs in the ROSI fetal tissue. (E) The expression level of genes located in the hypomethylated DMRs in the ROSI placental tissue. (F) The expression level of genes located in the hypermethylated DMRs in the ROSI placental tissue. [file Image_7.tif]

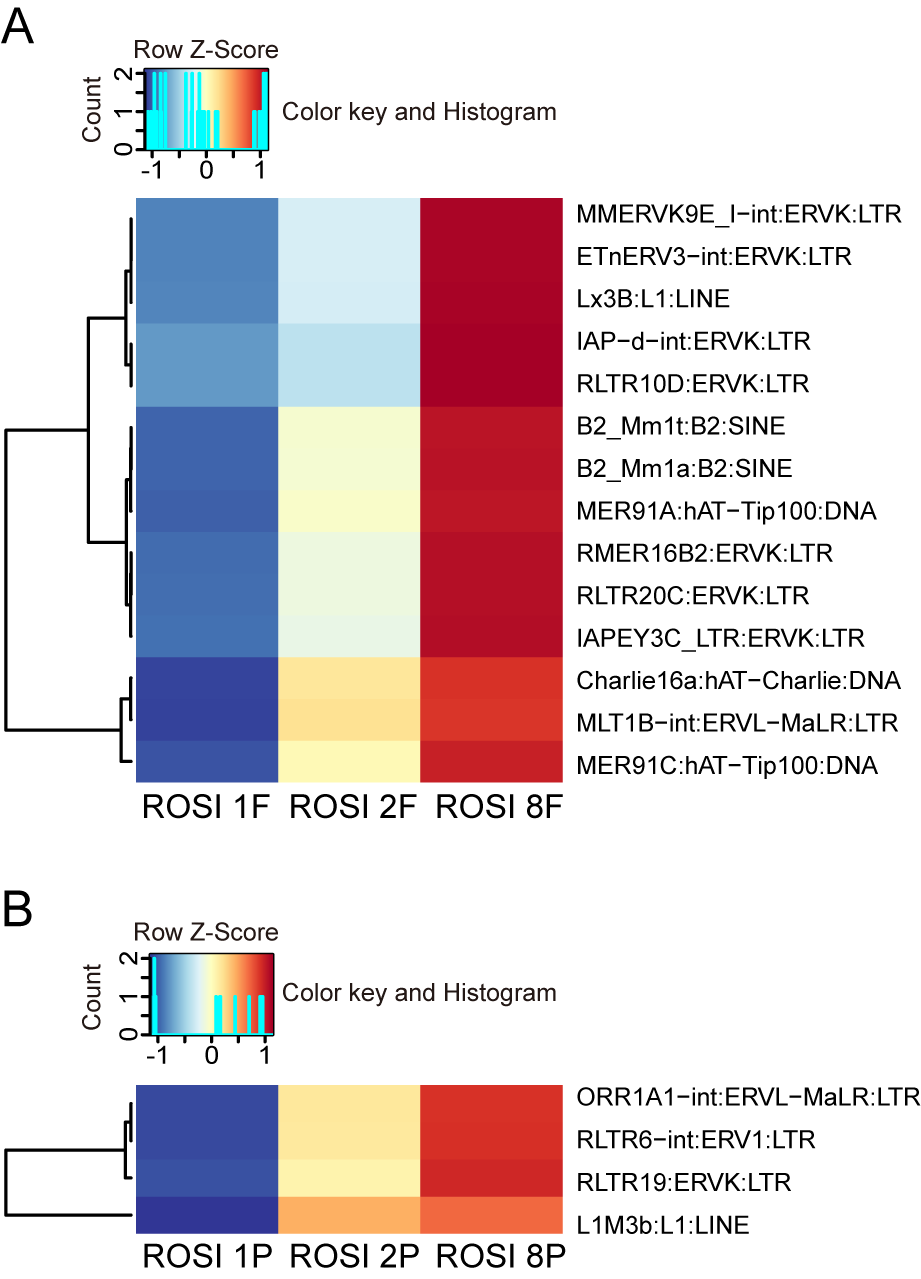

Supplement: Supplementary Figure 8 — Repetitive sequence (RS) expression in ROSI-derived fetuses and placentas with abnormal crown-rump length. (A) Heatmap depicting RS expression in normal and abnormal crown-rump length (CRL) fetuses. (B) Heatmap depicting RS expression in normal and abnormal CRL placentas. [file Image_8.tif]

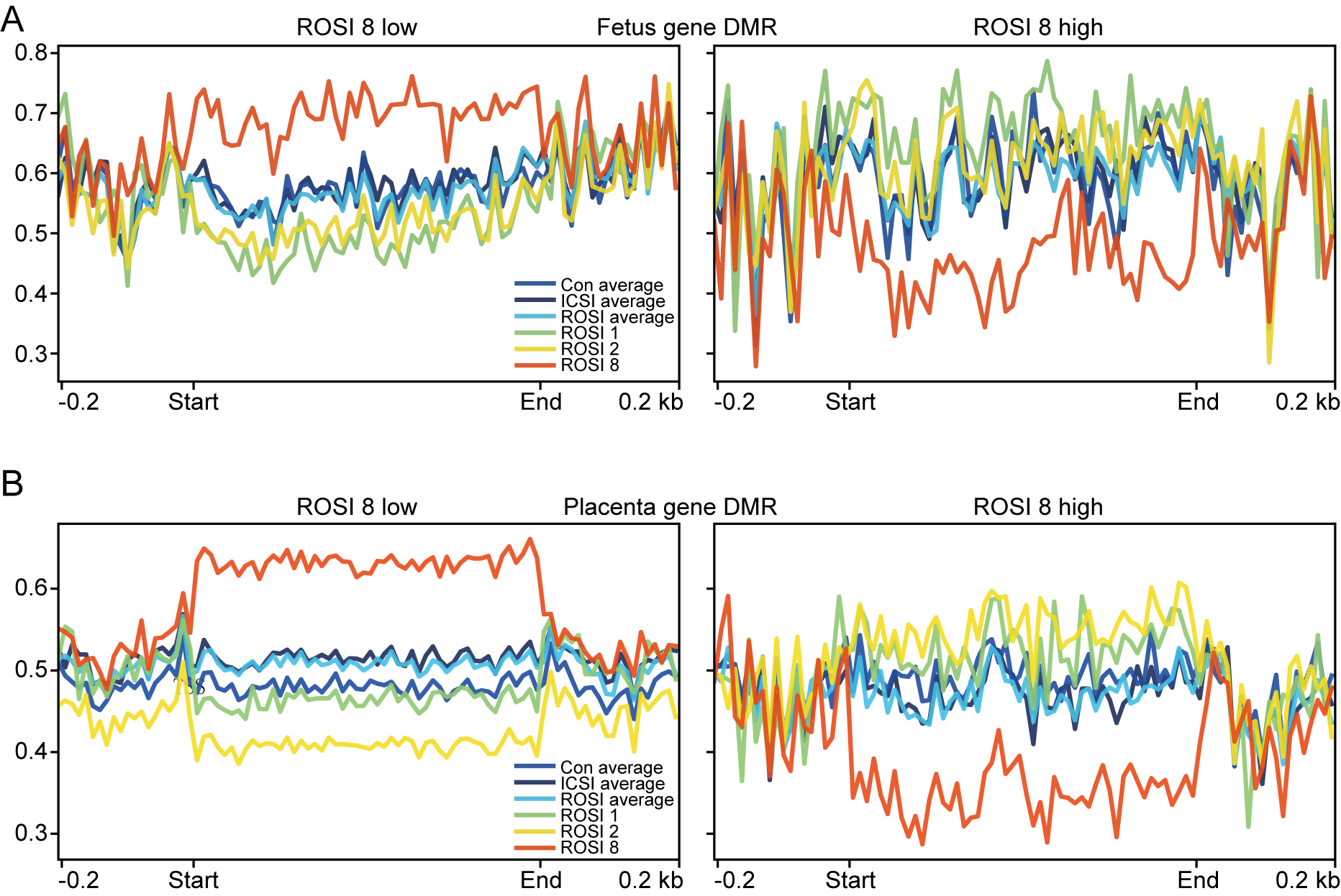

Supplement: Supplementary Figure 9 — DNA methylation status of DMRs between fetuses and placentas with normal and abnormal CRL. (A) DNA methylation status of DMRs between normal and abnormal CRL fetuses. (B) DNA methylation status of DMRs between normal and abnormal CRL placentas. [file Image_9.tif]
