# Supplementary material for: Transcriptome and DNA Methylation Profiles of Mouse Fetus and Placenta Generated by Round Spermatid Injection
Source: Front Cell Dev Biol. 2021 Mar 16;9:632183. doi: 10.3389/fcell.2021.632183 (PMC8009284; doi:10.3389/fcell.2021.632183)
Supplement: Supplementary file 10 [file Data_Sheet_1.docx]

**Supplementary Table 1. The sequence of primers used in RT-PCR**

| Category | Gene | Primer (5′→3′) | | Size (bp) |
| --- | --- | --- | --- | --- |
|  |  | Forward | Reverse |  |
| Differentially expressed genes in fetus | *Meg3* | CAGAGCGCTTCTGAAGACCA | CACCTACTGGGTGCTCACTG | 158 |
|  | *Malat1* | GAGCTCGCCAGGTTTACAGT | AACTACCAGCAATTCCGCCA | 97 |
|  | *Snora44* | TGCCTGTAGCCATGGGATCT | GTGCTGACCTGAGCTGTCAA | 70 |
|  | *Snora81* | GCACTGTGCTAAAATTGCAGG | GAGGTCCACCCCAGTCTTTG | 117 |
|  | *Snhg11* | AAGAGCCCTGCCTTCTGTTC | CAGTGTACACGAGCACAGGT | 153 |
|  | *Miat* | ACACCAACCCACAAGACCTG | GACAGATCAGCCATGTCCGT | 184 |
| Differentially expressed genes in placenta | *Ccl2* | TAAAAACCTGGATCGGAACCAAA | GCATTAGCTTCAGATTTACGGGT | 120 |
|  | *Ccl7* | CCAATGCATCCACATGCTGC | CTCGACCCACTTCTGATGGG | 164 |
|  | *Cdh13* | CTGTGGGGGTCATTGTCAACT | GTTGGTCTGTGGGTTGGTGT | 134 |
|  | *Ctss* | TAGAGGCAGACGCTTCCTATC | CGGGAGCTGAATGTACCTTGA | 101 |
|  | *H2-Eb1* | GCGGAGAGTTGAGCCTACG | AGGCCCGTGGACACAATTC | 165 |
|  | *Inhba* | TCACCATCCGTCTATTTCAGCA | CTTCCGAGCATCAACTACTTTCT | 134 |
|  | *Lzy2* | GAACGTTGTGAGTTTGCCAGA | TGCTCTCGTGCTGAGCTAAA | 100 |
|  | *Msr1* | AAGTATCAGCAGAAGTCCAGTCT | TCCTTCAGTCTGAGGTCGTTG | 112 |
|  | *Ang2* | AAGGAAGCCCTTATGGACGA | CCAGCCATTCTCACAGCCAA | 152 |
|  | *Rnase2a* | TGGAGCAACTTGAGTCTCGAC | CGGGGATAGGCTCTGTTATAGA | 130 |
| Imprinted genes | *Ascl2* | AAGCACACCTTGACTGGTACG | AAGTGGACGTTTGCACCTTCA | 115 |
|  | *H19* | GAACAGAAGCATTCTAGGCTGG | TTCTAAGTGAATTACGGTGGGTG | 106 |
|  | *Ifg2* | GTGCTGCATCGCTGCTTAC | ACGTCCCTCTCGGACTTGG | 222 |
|  | *Snrpn* | TGCTACGTGGGGAGAACTTG | CCTGGGGAATAGGTACACCTG | 156 |
|  | *Peg10* | TGCTTGCACAGAGCTACAGTC | AGTTTGGGATAGGGGCTGCT | 162 |
|  | *Ifg2r* | TGAATGGTGATCCTTGCCCTC | CCGGTAGCTGTTGGTCTGTC | 185 |
| Repetitive DNA sequences | ERVB4 | GCCTCAGCAGAGAGTGTGTT | GCATGGCTCCCTGTGTAACT | 125 |
|  | RLTR12A | CCAATGGCTGGGCAGAATAGA | GCTCTTTGGGCTTCAGGTGT | 168 |
|  | RLTR16B | AAGGCTGTGGGGGTTTTGAA | GCAATGGCCGACCTGTTTTT | 132 |
|  | RLTR20B3 | CAGGACAGAGAGCATAGCCG | GGCCACTTCCTAACCTCCAC | 164 |
|  | RLTR20C2 | GTAGGACGCAGAGAGGAACG | GGGCACTCCTAAACCTTCCC | 169 |
|  | MTE2b | GCTGAGCACCAGCATTCATC | GCTCACAGTTCGAGGGTACA | 121 |
| Internal reference | *Gapdh* | GACAAAATGGTGAAGGTCGGT | GAGGTCAATGAAGGGGTCG | 120 |
